# Recent Developments in Fermented Cereals on Nutritional Constituents and Potential Health Benefits

**DOI:** 10.3390/foods11152243

**Published:** 2022-07-27

**Authors:** Jiayan Zhang, Mengting Liu, Yansheng Zhao, Ying Zhu, Juan Bai, Songtao Fan, Lin Zhu, Ci Song, Xiang Xiao

**Affiliations:** 1 School of Food and Biological Engineering, Jiangsu University, Zhenjiang 212013, China; jiayanzhang1988@163.com (J.Z.); liumengting2000@163.com (M.L.); zhaoys@ujs.edu.cn (Y.Z.); ying307@126.com (Y.Z.); 1000005134@ujs.edu.cn (J.B.); fansongtao@ujs.edu.cn (S.F.); zhulin19820402@ujs.edu.cn (L.Z.); 15755082683@163.com (C.S.); 2 Inspection Quarantine Bureau Inspection and Quarantine Technology Center, Zhenjiang 212000, China

**Keywords:** fermentation, cereal, strain, nutrition, health benefits

## Abstract

Fermentation is one of the most economical and safe methods to improve the nutritional value, sensory quality and functional characteristics of raw materials, and it is also an important method for cereal processing. This paper reviews the effects of microbial fermentation on cereals, focusing on their nutritional value and health benefits, including the effects of fermentation on the protein, starch, phenolic compounds contents, and other nutrient components of cereals. The bioactive compounds produced by fermented cereals have positive effects on health regulation. Finally, the future market development of fermented cereal products is summarized and prospected.

## 1. Introduction

The Food and Agriculture Organization (FAO) estimates that by 2050, the global population will reach 9 billion [1]. In recent years, several major drivers have put the world off-track to ending world hunger and malnutrition in all its forms by 2030 [2]. The challenges have grown with the COVID-19 pandemic and related containment measures. Moreover, global warming poses an impending threat for global food security [3]. Thus, the problems of food supply can be effectively addressed by minimizing food loss and recycling waste, evaluating by-products, improving nutritional value and extending storage time. Fermentation, as an economic, universal and mature technology, can release the nutritional ingredient of underutilized cereals and improve their nutritional content to develop new sustainable foods with higher nutritional value [4].

Cereal is a traditional staple food of human beings and the main source of carbohydrates in food [5]. Cereals provide large amounts of energy, protein and micronutrients in animal and human diets. They are considered to be one of the most important sources of dietary protein, carbohydrates, vitamins, minerals and fiber for people around the world. Cereals contribute around 50% of the mean daily energy intake in most populations, and 70% in some developing countries [6]. However, most cereals are more or less deficient in some essential nutrients, such as the essential amino acids threonine, lysine and tryptophan. On the other hand, cereals contain certain anti-nutrients, such as phytic acid, tannin and non-starch polysaccharides. Anti-nutritional factors have low digestibility due to their easy binding with proteins [7]. Furthermore, plant-based proteins usually contain fibers that hinder the access of proteases, which therefore decreases protein digestibility [8].

Food fermentation dates back hundreds of years and is considered a food preservation technique, as well as a tool for obtaining new flavors, fragrances and textures [9]. Fermentation can be defined as the biological process by which microorganisms transform substrates into new products, such as enzymes, and primary and secondary metabolites [10]. In the microorganisms used for cereal fermentation, we often use molds or fungi, bacteria and yeast [11]. Changes in fermentation conditions contribute to enzyme activation, and changes in pH can improve the performance of certain enzymes, such as amylase, protease, hemicellulase and phytase. Enzyme-induced changes and microbial metabolites work together to give fermented cereal foods good process and nutritional effects [12]. As a unit operation in food processing, fermentation offers a large number of advantages, including: improving food safety, flavor and acceptability, increasing variety in the diet, improving nutritional value, and reducing anti-nutritional compounds [13,14]. Many papers have reviewed the changes in the nutrient composition of cereals caused by fermentation, but they did not distinguish between different cereals. Therefore, this paper reviews the effects of different fermentation strains and fermentation techniques on different cereals, including the changes in their nutrient content and the health benefits of fermented products. Moreover, the future market development of fermented cereal products is summarized and prospected.

## 2. Modern Fermentation Technology

**Figure 1:**
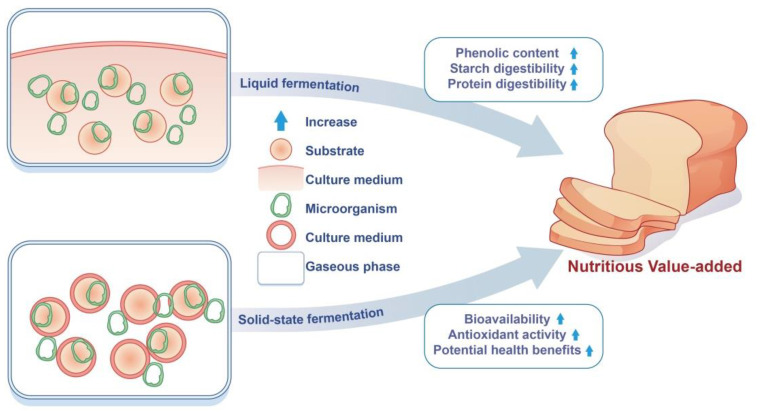
Comparison between liquid and solid-state fermentations.

According to the state of the fermentation system, the microbial fermentation process can be divided into liquid fermentation (LF) and solid-state fermentation (SSF) (Figure 1). Liquid fermentation is based on the cultivation of microorganisms in a liquid medium containing nutrients. Solid-state fermentation involves the growth and product formation of microorganisms on solid substances in the absence (or near absence) of free water [15].

### 2.1. Solid-State Fermentation Technology

Solid-state fermentation, as a biological process, can not only achieve large-scale production and maximize the value of cereals in a simple way, but also reduce investment and production costs, and does not produce a large amount of wastewater. It is an important means of energy conservation, emission reduction and low-carbon production at present [16,17]. In recent years, with the deepening of related research, solid-state fermentation has been widely recognized and applied. In solid-state fermentation of wheat bran with *Bacillus* TMF-2, phytic acid was partially degraded, with a maximum degradation rate of 34% as the fermentation went on. Furthermore, the content of soluble phenols is almost three times that of raw wheat bran. The total proportion of polyphenols, antioxidant capacity and the free radical scavenging rate were significantly increased; in particular, the reduction capacity of Fe^3+^ increased 10-fold [18].

### 2.2. Liquid Fermentation Technology

Liquid fermentation mainly makes materials into a liquid state at first, and then inoculating strains after sterilization, providing enough oxygen and a suitable external environment, so that the strain can multiply in large quantities. The advantages of liquid fermentation are a wide range of raw materials, fast bacterial growth and short production cycle. Moreover, liquid fermentation can effectively reduce the rate of bacterial contamination, and can be industrialized and is not limited by the season [19]. It has been reported that a maximum 23.99 mM of ferulic acid was released from rice bran by *Pediococcus acidilactici* in the liquid fermentation process [20].

## 3. Fermentation Strain

The common fermenting bacteria are species of *Lactobacillus*, *Bacillus*, *Pediococcus*, *Micrococcus* and *Streptococcus* [21] (Table 1). In cereal fermentations, species of *Lactobacillus* and *Bacillus* play a significant role in fermentation, as they hydrolyze complex polyphenols to simpler ones and form biologically active compounds [22].

Yeast fermentation is commonly used in the preparation of alcoholic beverages, but now yeast is also being evaluated for its potential to enhance cereals. Since yeast can grow in a substrate with a low water content, solid-state fermentation can be carried out using raw materials such as wheat bran and rice bran [49]. The common fermenting yeasts are species of Saccharomyces, which results in alcoholic fermentation. A study found that fermentation with yeast improves the cereal flavor and increases the antioxidant capacity of cereals [50].

The common fermenting fungi are *Aspergillus*, *Paecilomyces*, *Cladosporium*, *Fusarium*, *Penicillium* and *Trichothecium*. In addition, food-grade filamentous fungi (*Aspergillus* sp. and *Rhizopus* sp.) are used to increase the amount of free polyphenols, so as to enhance the bioactivity of various foods [51]. These fungi also produce highly digestible proteins without any toxic substance being generated [52].

Single-strain treatment of cereal often has the defect of low fermentation efficiency, which limits its application in fermentation. Co-fermentation of two or more microorganisms is used to achieve the synergistic effect of metabolism of mixed culture strains [53]. In recent years, microbial mixed fermentation has shown a series of advantages, which can share the metabolic burden through the division of labor between strains and has the ability to effectively transform complex substrates. For example, in the co-culture of filamentous fungi *Aspergillus Niger* and *Aspergillus oryzae* in wheat bran, the two strains were evenly distributed, and the mixed culture secreted more enzymes that degrade the cell wall [54]. *Monascus* and *Bacillus subtilis* were co-cultured, the total phenol content of fermented oat was 23 times that of unfermented oat [55]. Sourdough inoculated with *Bifidobacteria* was able to increase the phytate hydrolysis and raise organic acid levels that modify the starch digestibility, which could contribute to lowering the glycemic index [32].

## 4. The Changes in Nutrient Composition

**Figure 2:**
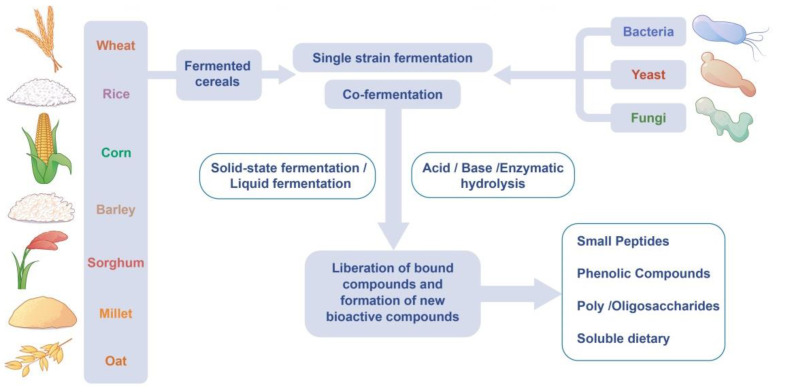
The release and production of bioactive compounds by fermentation.

Research in recent years has shown that fermented cereals have great potential to improve and expand their health benefits. Fermentation can increase the biological activity of cereals, and it helps to improve the ratio of nutritional composition to anti-nutritional components, which encourages the production of new functional foods [56] (Figure 2). In this section, we focus on the effects of fermentation on the main functional components of cereal (Table 2).

### 4.1. Changes in Nutrient Composition of Fermented Wheat

Wheat (*Triticum aestivum* L.) is one of the most important grains consumed by humans and a major source of energy [74,75]. Wheat and gluten-containing products have been linked to a range of intestinal diseases, reducing their consumption worldwide and leading to considerable demand for gluten-free products [76]. Fermentation can improve the nutritional value of cereals and the sensory quality of products, and fermented products are suitable for a wider range of people to eat [77].

The surface of wheat starch granules was slightly eroded during natural fermentation. Pasting properties of wheat starch were changed by natural fermentation. The results show that fermentation is an effective method to modify wheat starch, and it has the potential to improve the quality of starch-based food [57]. Ge et al. studied the effect of co-fermentation of *Lactobacillus plantarum* and *Saccharomyces cerevisiae* on the structure and flavor of wheat noodles. They found that co-fermentation enhanced the continuity of the gluten network and promoted the formation of pores. Co-fermentation significantly increased the α-helix ratio of gluten protein and enhanced the order of the protein molecular structure. Wheat noodles were endowed with more desirable volatile components [58]. Moreover, Antognoni et al. used different lactic acid bacteria strains to ferment wheat. Fermentation promoted the release of phenolic substances, especially ferulic, p-coumaric, cinnamic, caffeic, sinapic, p-hydroxybenzoic and gallic acids in the process of fermenting wheat [24]. A similar result was found by Muhammad et al.: when wheat was fermented for 72 h, the content of free phenolic compounds increased significantly [28]. Tannin is rich in wheat, corn, barley and millet, which can inhibit the activity of digestive enzymes, reduce protein decomposition and affect food intake. Some scholars believe that lactic acid bacteria fermentation can hydrolyze tannins and phytic acid in cereals [78]. The degradation rate of phytic acid and tannins of wheat anti-nutritional factors reached its maximum after 72 h spontaneous fermentation, in which the content of tannins decreased from 0.58 mg/100 g to 0.47 mg/100 g, and the phytic acid content decreased from 464.1 mg/100 g to 371.28 mg/100 g [59].

Wheat bran is a cheap lignocellulosic biomass and the main by-product of wheat flour production [79]. Wheat grain consists of bran, germ and endosperm. The bran is located in the outermost layer of the wheat grain, accounting for about 13% to 19% of the total weight of wheat grain [80]. Wheat bran contains a large amount of total dietary fiber (451 g/kg), as well as other related compounds, including protein (160 g/kg), fat (47 g/kg), carbohydrates (177 g/kg) and minerals (61.5 g/kg) [81]. It appears to be an abundant and cheap material for the production of functional food and feed ingredients. However, wheat bran contains more phytic acid and other anti-nutritional factors. Besides, the content of insoluble dietary fiber is higher [82].

Arte et al. analyzed the modification effect of bioprocessing on wheat gluten; they found fermentation degraded the protein into amino acids and peptides, while increasing the content of free amino acids. In addition, there is a clear tendency of increasing phytase activity and decreasing anti-nutritional factors [37]. Zhao et al. used commercial baker’s yeast and lactic acid bacteria to ferment wheat bran. The co-fermentation of the two microorganisms reduced phytic acid levels by 27.34%, degraded cell wall components and produced more flavor compounds. Moreover, the total dietary fiber and soluble dietary fiber increased after solid-state fermentation [35]. The non-starch polysaccharides in cereals are difficult for the digestive system of single-stomach organisms to digest and absorb. Furthermore, they tend to cause the accumulation of digesta in the digestive tract, and lead to intestinal microbial disorder [65]. Studies have shown that *Bifidobacteria* can use xytritose and xytetracose [83]. In addition, fermentation of wheat bran with *Bacillus* can effectively reduce the content of non-starch polysaccharides [31]. Therefore, fermentation can improve the bioavailability of nutrients and the content of bioactive substances.

### 4.2. Changes in Nutrient Composition of Fermented Rice

Rice (*Oryza sativa* L.) production is concentrated in Asia (around 90% of total world production), with China and India being the largest national producers and consumers of rice. It is also considered a promising ingredient in gluten-free products that are nutritious, easy to digest, and hypoallergenic, attracting widespread attention from the food industry and researchers [84]. However, rice flour lacks viscoelastic gluten protein, has lower viscosity, and the processed product has an unsatisfactory appearance, taste, aroma and texture [85]. Rice is considered to be a high glycemic index (GI) food [86]. Long-term consumption of high GI foods can lead to chronic diseases such as obesity, cardiovascular disease and type Ⅱ diabetes [87,88]. Microbial fermentation is one of the necessary processes for the fermentation of rice cake and rice flour, which can better control the function of rice-based foods [84].

Research was also conducted by Li et al. to evaluate the effect of *Lactobacillus fermentum* M9 and *Candida santamariae* Y11 co-fermentation on rice. The fermentation disrupted the ordered structures of rice (starch crystallites) and broke starch granules, which was preferable for the swelling and molecule leaching of rice noodle matrixes with enhanced molecule interactions [38]. The contents of total starch and amylose in rice flour were significantly increased by fermentation of rice flour for 5–10 days. Fermentation degrades the amorphous region of starch grains by enzymes and organic acids, which reduces the polydispersity index and molecular weight of the molecular structure of starch, thus reducing the cooking loss of extruded instant rice [89]. The same results were obtained by fermentation of rice starch with *Yeast* and *Lactobacillus* strains [60]. A study reported that the structure of rice starch did not change significantly, but the content of protein decreased after 72 h of natural fermentation. The characteristics of gelatinization enthalpy and texture were changed. After fermentation, the contents of hydrophobic amino acids in rice flour increased. They found that the substantial effect of fermentation on the functional properties of starch was related to protein properties [90]. Fermentation caused a significant increase in the extractable total of phenols in fermented brown rice flour of 13%. In addition to this, the contents of zinc, phosphorus, magnesium, iron and calcium increased significantly, while phytic acid decreased by 41% after fermentation. The reduction in pH during fermentation may activate phytase that degrades phytic acid complexes and releases minerals. The results indicate that fermentation increases mineral bioavailability and decreases anti-nutrients [61].

Rice bran is a by-product produced during rice milling, accounting for about 10–13% of brown rice [91]. Rice bran is rich in nutrients and bioactive ingredients, including dietary fiber, vitamins, ferulic acid and γ-aminobutyric acid, tocopherol and vitamin E [92]. Rice bran is an important nutrient source of oil, protein and non-starch polysaccharide, and has more nutrients than white rice [93]. However, rice bran has been mainly used as animal feed, which is a huge waste of food resources [94]. Food-processing technology such as fermentation can improve the sensory quality of rice bran [95]. Proteins increased more than two times when compared to the unfermented bran at 0 h; the content of protein increased from 11% to 29% after 4 days of fermentation [96]. With respect to the effect of fermentation on phenolic compounds, Chen et al. reported an increase of free phenols and bound phenols in rice bran caused by *Rhizopus oryzae* [97]. Ranjan et al. inoculated *Rhizopus oryzae* in de-oiled rice bran; the trypsin inhibitor activity significantly decreased by 24.8% and phytic acid activity decreased by 3.12% after 3 days of solid-state fermentation [63].

### 4.3. Changes in Nutrient Composition of Fermented Corn

Maize or corn (*Zea mays* L.) is the main cereal crop grown and produced on all five continents [98]. Corn is considered the third most important cereal crop in the world after wheat and rice, and is one of the population’s staple foods [81]. In respect to nutritional quality, corn can not only provide sufficient energy for the human body, but it is also rich in protein, minerals, vitamin lecithin, calcium, magnesium, selenium and other trace elements. Corn can enhance the human metabolism and adjust the function of the nervous system. Moreover, corn plays a positive role in the prevention of fatty liver, stomach disease, enteritis, skin aging and even cancer [99]. Corn lacks gluten. It is difficult to form gluten, resulting in easy water loss and cracking of dough, poor viscoelasticity and extension. In addition, pure cornmeal food has a poor taste, so a lot of corn is used to make alcohol or feed, and only about 5% of corn is grown directly for food [100]. Therefore, corn flour was modified by means of microbial fermentation to improve bioavailability.

The work of Yaqoob et al. showed the effect of lactic acid bacteria (LAB) on corn flour. During the fermentation, Lactic acid bacteria hydrolyzed part of the corn flour, resulting in smaller, irregularly shaped particles with more holes in them. Furthermore, the textural, thermal and pasting profile was also improved due to the degradation of macromolecules, making it more suitable for processing various flour products than before fermentation [64]. *Lactobacillus plantarum* T6B10 and *Weissella confusa* BAN8 were used as selected starters to ferment corn milling by-products. After fermentation, the content of free amino acids and polypeptides improved. Adding fermented corn by-products to bread significantly improves protein digestibility (up to 60%) [101]. Salar et al. found that, with the increase of total phenolic content in raw materials, the activity of β-glucosidase was enhanced in the process of corn fermentation, which could hydrolyze phenolic glycosides and release free phenols [48]. The reason may be that enzymes produced in the process of microbial fermentation promote the modification of cereal and the distortion of chemical bonds, thus accelerating the further degradation of bound phenols [51]. Decimo et al. fermented corn bran with *Lactobacillus plantarum*, which increased the content of soluble dietary fiber in corn, while reducing the content of phytic acid, which is conducive to the body’s absorption of calcium, magnesium and other important elements [102]. Sokrab et al. also found a similar phenomenon: the change in the content of trace elements in corn meal fermented by lactic acid bacteria was related to the change of mineral content during fermentation [103]. Tchikoua used lactic acid bacteria to ferment corn meal. They found that tannins, phytic acid and non-starch polysaccharides were reduced, improving its nutritional properties [83].

### 4.4. Changes in Nutrient Composition of Fermented Barley

As the fourth largest cereal in the world, barley (*Hordeum vulgare* L.) grains are rich in dietary fiber, protein, minerals and a variety of other bioactive phytochemicals [104]. Eating whole barley helps control weight and reduces the risk of chronic diseases such as heart disease, type Ⅱ diabetes and colon cancer [105,106]. However, the sensory quality of barley was poor due to its high dietary fiber content and low gluten protein content [107]. The majority of barley production has been used for animal feed and brewing material; only about 10% of barley is consumed directly by humans in China, leaving a lot of waste [108,109]. Fermentation, as a biological processing method, is one of the effective and mild treatment methods for improving the properties of the raw materials of food.

Using natural fermentation of barley starch decreased the amount of amylopectin long chains, while increasing short chains. They found that after 72 h of fermentation, grains with more pores, that were broken and cracked, were found under the microscope, and their molecular weight decreased from 2.26 to 1.04 ×10^8^ g/mol [66]. Xiao et al. found that the proportion of essential amino acids such as glutamate, glycine, alanine and methionine in fermented barley powder increased [67]. This may be related to the mutual transformation of amino acids during *Lactobacillus plantarum* fermentation. Lactic acid bacteria produce proteases during fermentation, which hydrolyze proteins, increase the content of free amino acids [110]. Bamdad et al. found the same result when they studied the fermentation of barley protein by lactic acid bacteria [111]. Zhang et al. found that the content of free phenolic acid, especially the content of ferulic acid, increased significantly after barley fermentation [112]. The reason is that lactic acid bacteria can secrete ferulic acid ester enzymes; these esterase hydrolyze carboxylate bonds with xyllase, and release polyacid compounds such as ferulic acid from polysaccharide [22]. Xiao et al. found that the structure of β-glucan could be changed by fermentation, the molecular weight of β-glucan was reduced, and the proportion of β-(1→3) and β-(1→4) residues increased. These structural changes enhanced the water adsorption or molecular binding ability in barley [68]. Fermentation is an important means of modification and release in cereal dietary fiber. Due to the production of organic acids, a variety of endogenous enzymes or bacterial enzymes in cereal may be activated, biopolymers are degraded, the texture softens, and fiber is dissolved or released [113].

### 4.5. Changes in Nutrient Composition of Fermented Sorghum

Sorghum (*Sorghum bicolor* L.) is the fifth largest cereal in the world, after wheat, rice, corn and barley, and plays an extremely important role in agricultural production [114]. Sorghum is a staple food for people and livestock around the world, especially in arid areas of Africa and Asia [115]. Although sorghum has contributed to global food production, the value of sorghum has not been fully utilized due to its poor palatability and rough taste, which is not easily accepted by consumers [116]. Sorghum grains are rich in antioxidants such as polyphenols, which may protect against certain cancers, prevent diseases related to oxidative stress, antibacterial and anti-inflammatory effects, and it also improves glucose metabolism [117].

Starch is the main carbohydrate in sorghum; the mass fraction is about 60~70%, and the highest can reach about 80%, so the physical and chemical properties of starch directly affect the food quality of sorghum [118]. Ge et al. researched natural fermentation of sorghum. They found that fermentation can increase the content of sorghum amylose. After fermentation, the gelatinization temperature of sorghum starch decreased and its physical and chemical properties changed. The aging characteristics of starch were improved, and it was more suitable for the production of products made from starch aging characteristics [69]. The average crude protein content of sorghum was 9.36%, ranging from 8.26% to 10.46% [115]. The strong bonds between sorghum protein and starch limits the hydrolysis and effectiveness of nutrients [119]. In addition, the existence of anti-nutritional factors can interfere with the digestion of protein in grains. Therefore, sorghum protein has the disadvantage of low digestibility and poor quality [120]. Mohapatra et al. used lactic acid bacteria (LAB) to ferment sorghum grains. It was observed that both the essential and non-essential amino acids increased during the LAB fermentation, while the anti-nutritional factors such as trypsin inhibitor also decreased with fermentation. Moreover, they also found the content of crude fibers increased from 2.76% to 3.41% during fermentation. Fermented sorghum can improve the bioavailability of nutrients, especially amino acids [70]. Studies have also shown that *Lipomyces kononenkoae* and *Saccharomyces cerevisiae* fermented sorghum, both of which reduced the phytic acid content and the ratio of phytic acid to protein, thus improving the digestibility of sorghum protein [26]. Similar results have been reported by ELKhier [121].

### 4.6. Changes in Nutrient Composition of Fermented Millet

Millet (*Setaria italic* var. *germanica* (Mill.) Schred) has a short growing period and strong resistance to pressure, and its grains are easy to store. China leads the world in millet production, accounting for four-fifths of total production. Millet is a gluten-free, nutritious whole grain food. Compared with our current staple foods of rice and wheat, millet contains a variety of vitamins, minerals and a high level of protein [122]. The proportion of nutrients is reasonable, and the body can make good use of these nutrients. In addition to being nutrient-rich, it also has a lower glycemic index than staple foods made from rice and wheat flour [123]. Furthermore, some polyphenols in millet have antioxidant properties and have been associated with reduced risk of chronic diseases and oxidative stress responses [124]. However, millet is an underutilized food resource in many countries, so it has great potential as a food and beverage source for humans [125]. Fermentation produces a variety of metabolites, including antioxidants, vitamins and polyols, which may confer specific health benefits [126].

The amylose content of glutinous proso millet starch fermented by *Lactobacillus plantarum* increased and then decreased, and the distribution of amylopectin chain length tended to be short-chain. Fermentation can improve the properties of starch, especially water absorption, expansion and gelatinization, which is helpful for starch modification [71]. It was found that the total phenolic content (TPC) was enhanced after fermentation. Fermentation by *Rhizopus azygoporus* significantly increased the TPC in pearl millet from 6.6 to 21.8 mg, which was due to the release of phenolics through the activity of carbohydrate lyase and β-glucosidase [44]. Fermentation was further observed to increase the majority of essential and non-essential amino acids. Therefore, fermentation of pearl millet increased the nutritional value of flour, which is a potential material for gluten-free products [72]. *Bacillus. natto* fermentation enhanced the soluble dietary fiber content from 2.3% to 13.2%, and the soluble dietary fiber/insoluble dietary fiber ratio from 3.1% to 19.9%. During fermentation, the cellulose and hemicellulose degraded, thereby forming more porous and loose structures and polysaccharides in millet bran [30].

### 4.7. Changes in Nutrient Composition of Fermented Oat

Oat (*Avena sativa* L.) is an annual herb, one of the eight major cereal crops [127]. Oat is unique among all cereal crops because of its high concentration of dietary fiber, β-glucan, unsaturated fatty acids and phenolic compounds [128]. In addition, due to the highest protein content (12.4–24.5%) and balanced amino acid composition, oats are considered as a potential low-cost protein source and food material to replace meat and dairy products [129,130]. The polyphenols found in oats include phenolic acids (such as caffeic acid, p-coumaric acid and ferulic acid), flavonoids and a unique group of amides called oat anthramide, which have a strong antioxidant capacity in vitro and in vivo [42]. Therefore, oats have a high nutritional value for human food, animal feed and health care [131].

Research shows that after solid-state fermentation with *Lactobacillus plantarum* and *Rhizopus oryzae,* the soluble protein contents changed from 7.05 mg/g to 14.43 and 10.21 mg/g for the co-inoculated fermented oats (CFO) and the *R. oryzae*-fermented oats (RFO), respectively. In addition, both CFO and RFO presented higher ACE inhibitory activities than unfermented oats [73]. It was found that oat protein was hydrolyzed into polypeptides and small molecular proteins after solid-state fermentation with *Lactobacillus plantarum* and *Bifidobacterium animalis*; the nutritional value and antioxidant activity of oat protein were also significantly enhanced [33]. The reason may be that the pH value of the substrate decreases after fermentation, the endogenous protease is activated, and the macromolecular protein is degraded [110]. On the other hand, due to hydrolysis in fermentation, the interaction with protein may be weakened and the solubility of protein increases [132]. Calinoiuc et al. found that the content of avenanthramide and ferulic acid in oat bran increased by 48.5% and 21.2%, respectively, after solid-state yeast fermentation [133]. This increase in free phenolic acids has previously been shown in other studies; the bioavailability of oat powder fermented by lactic acid bacteria was improved [25]. It was found that this may be related to the degradation of the ceral’s dense cell wall matrix by enzymes. Enzymes are derived from fermentation microorganisms that promote the release of phenolic compounds [134].

## 5. Health Benefits of Fermented Cereals

### 5.1. Antioxidant Activity

Microorganisms can modify antioxidant constituents during the fermentation process. In general, biological activity is assessed by different chemical tests including DPPH, ORAC, FRAP and ABTS for antioxidant and free radical scavenging in vitro. Indeed, various studies have shown that phenolic compounds are positively associated with antioxidant activity, and microbial fermentation can enhance the dissolution and extraction efficiency of total polyphenols [7,135,136]. For example, the contents of ascorbic, galli, and p-coumaric acids in pearl millet increased remarkably with fermentation, thus enhancing the antioxidant activity of pearl millet extracts [137]. Research showed that the total polyphenol content increased from 0.20 g/100 g in unfermented wheat bran to 0.81 g/100 g in fermented wheat bran. Meanwhile, the DPPH and ABTS free radical scavenging rate of wheat bran significantly increased after *Eurotium cristatum* fermentation [46]. Due to fermentation, free phenolic content in black barley increased to 5.61 ± 0.02 mg GAE/mL. Moreover, free phenolic extracts from fermented barley played a greater role in targeting against H_2_O_2_-induced oxidative injuries in human hepatocarcinoma cells, which inhibited ROS production and improved cell viability, cell membrane integrity and SOD activity [23]. Moreover, Chu et al. found that the content of total phenolic acid from millet bran increased. Fermentation enhanced the free radical scavenging ability of DPPH, and antioxidant activity increased after fermentation [30]. During the fermentation process, the content of antioxidant polysaccharides, antioxidant peptides and phenolic compounds increased through microbial hydrolysis or biotransformation. Overall, results indicate that fermentation in many cases contributed to enhancing antioxidants’ content and antioxidant capacity.

### 5.2. Antiobesity and Anti-Glycolipid Disorder

Obesity and its related metabolic syndrome have become a global challenge, placing a common burden on mankind. The development of obesity is caused by disturbance of lipid metabolism and glucose homeostasis, and these diseases are often accompanied by oxidative stress and insulin resistance [138,139]. Zhang et al. found that *Lactobacillus plantarum* fermentation of barley extract (LFBE) can regulate lipid metabolism and improve insulin resistance in obese rats through animal experiments. LFBE can significantly reduce body weight, decrease the contents of total triglyceride (TG) and total cholesterol (TC) in serum and the liver, and improve the glucose tolerance of obese rats [112,140]. In fact, the use of pre-fermentation technology (sourdough) could reduce starch digestibility to regulate glucose metabolism [12]. Gu et al. found that *Lactobacillus plantarum* fermentation of barley extract protein significantly increased glucose consumption. Moreover, *Lactobacillus plantarum* fermentation of barley extract protein significantly increased the expression of UCP1, PGC-1 and other genes directly related to lipid lowering and thermogenesis in 3T3-L1 cells [141,142]. These findings indicate that natural antioxidants, such as polyphenols, protein from fermented cereals, may be potentially used as functional food ingredients to prevent obesity and hyperlipidemia.

### 5.3. Anti-Inflammation Activity

Inflammation is the immune system’s response to harmful stimuli. Inflammation can be triggered by a variety of factors, including pathogens, damaged cells and toxic compounds [143,144]. Thus, inflammation is a defense mechanism that is critical to health. Studies have shown that polyphenols found in fermented products are beneficial to microbial metabolism and growth, and can inhibit the production of inflammatory cytokines [145,146]. YIN et al. isolated an *Aspergillus niger* strain from solid-state fermentation, and found that it significantly increased the release of bound ferulic acid in wheat bran. In addition, TNF-α, IL-6 and NO hydro levels indicated that fermentation of wheat bran could inhibit the inflammatory response induced by lipopolysaccharides [39]. Morena et al. used yeast and lactic acid bacteria to co-ferment wheat flour, and the fermented wheat flour showed higher antioxidant activity. In the transfected HT-29 cells, the fermentation broth effectively protected TNF-α-induced changes by significantly reducing the expression of IL-8 and COX-2 inflammatory mediators, which has a potential role in the treatment of intestinal inflammatory diseases [147]. The extract of barley fermented by *Aspergillus* can reduce chronic alcoholic liver injury and inhibit the proliferation of inflammation by inhibiting oxidative stress [148,149]. Moreover, fermented black rice bran extracts (FFs) were administered to rats with alcohol-induced chronic liver injury over 12 weeks. Treatment with FFs was also found to normalize the alcohol-induced upregulation of gene expression on critical inflammatory markers [150].

### 5.4. Anti-Cancer Activity

Various components in the grain, such as binding phenols, free phenols, free polyphenols, β-glucan, have cytotoxic effects on a variety of cancer cells [151]. Functional substances with antioxidant and immunomodulatory in cereals are associated with an anti-cancer effect [152]. Xiao Xiang et al. found that the *Lactobacillus plantarum* fermentation of barley extract (LFBE) can significantly inhibit human colon cancer HT-29 cell proliferation, which has the effect of inducing apoptosis of the cancer cells [153]. Japanese scholars found that residues from *Saccharomyces cerevisiae* fermentation of barley Shochu had therapeutic effects on mice models of liver cancer cells. Furthermore, the study demonstrated that fermentation products have stimulation effects on anti-tumor immunity. Suphot et al. investigated the chemo-preventive effect of fermented brown rice and rice bran (FBRA) on inflammation-related colorectal carcinogenesis in ApcMin/+ mice. These results suggest that FBRA significantly suppressed the multiplicity of colon tumors in comparison with the control diet group [154]. Additionally, Kunishige et al. found that fermented brown rice and rice bran with *Aspergillus oryzae* is an effective chemo-preventive agent against inflammation-related carcinogenesis that acts by inhibiting inflammatory cell infiltration into inflammatory lesions [155].

### 5.5. Immunomodulation Activity

Immunity is critical to maintaining homeostasis, which keeps animals healthy and growing. The destruction of the immune system is the cause of various diseases. For example, cancer progresses by lowering immunity [156]. In other words, cancer growth seems to be effectively suppressed by boosting immune activity. Wang et al. used *Bacillus subtilis* and *Saccharomyces cerevisiae* to ferment wheat bran polysaccharides. The fermented wheat bran polysaccharides affect the immune responses of lambs, inducing the production of IgG and IgM. The results show that fermented wheat bran polysaccharides could stimulate the secretion of anti-inflammatory cytokines [157]. Wang et al. found that long-term supplementation of 10% fermented wheat bran can improve the immune performance and laying performance of laying hens by affecting serum biochemistry, reproductive hormones and inflammatory responses [158]. Other researchers have also found the same conclusion; they used *Bacillus*, *Lactobacillus* and *Saccharomyces cerevisiae* to ferment corn, soybean and wheat bran as chicken feed. After long-term feeding, fermented diets improved the intestinal morphology and barrier function of laying hens [159]. The mechanism is complex and may be related to more nutrients produced by fermented cereals. In addition, fermented cereals have better anti-inflammatory effects and can improve gastrointestinal immunity. These all help to improve the body’s immune function.

## 6. Conclusions

**Figure 3:**
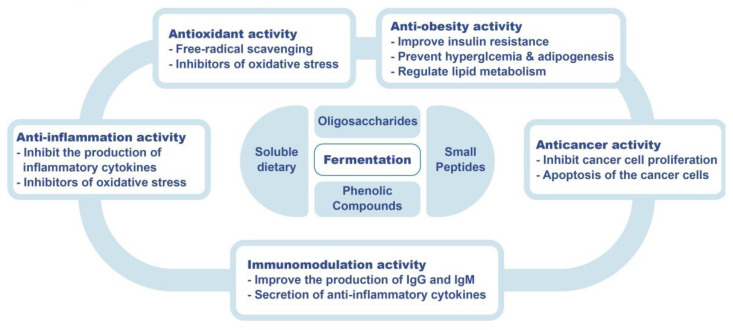
Schematic overview of possible beneficial health mechanisms of fermented cereals.

This review explores the potential of microbial fermentation in cereal science, showing its positive impact on food nutrition and health. The added nutritional value of fermentation improves the properties of the product, making it a better food ingredient than the original cereal. To sum up, fermentation is considered to be an effective tool to enhance the nutritional and functional value of cereal products, meeting the needs of modern consumers for health-promoting products and bringing new opportunities to the food industry (Figure 3). Most of the processing technologies of fermented cereals food are still in the laboratory stage and a long way from large-scale industrialization and industrial production. Accordingly, further optimization of the processing technology and formula to meet the needs of industrialized production has also become an important direction of research on fermented cereals food processing. Future efforts should focus on industrial production, research on fermentation technology, and achieve large-scale production. In addition, current studies on the health benefits of cereal fermentation products are limited to mice and other animals, and there is still a lack of adequate clinical validation. Therefore, further clinical trials need to be designed to reveal the fundamental mechanism of action of cereal fermentation products on human health. Cereal-based fermented products are often limited by the sensory characteristics of the products, making it a challenge to strike a balance between health and good flavor to satisfy the tastes of consumers around the world. The future viability and success of these fermented cereal products depends on their consumer acceptance. Therefore, further research on the innovations of fermented foods is encouraged, including the fermented foods currently on the market. This review has provided helpful information on the effect of the fermentation process on the bioactive substances and health benefits of different cereals.

## Figures and Tables

**Table 1:** List of Fermentation Strains of fermentation cereals.

Category	Microorganism	Material	Results	References
Bacteria				
	*Lactobacillus plantarum* (P-S1016)	Barley	The free phenolic content ↑ The phenolic composition ↑	[23]
	*Lactobacillus fermentum* (MR13), *Lactobacillus rhamnosus* (C249, C1272), *Lactobacillus plantarum* (LB102, LB124, LB126, LB245, 29DAN, 83DAN, 6BHI, 98A) *Lactobacillus brevis* (3BHI)	Wheat	The phenolic compounds content ↑	[24]
	*Lactobacillus acidophilus* (LA-5), *Lactobacillus johnsonii* (LA1), *Lactobacillus reuteri* (SD2112)	Oat Barley	The content of free phenolic acids ↑	[25]
	*Lactobacillus amylovorus* (NRRL B-4540)	Sorghum	The anti-nutritional factors content ↓ The digestibility of protein ↑	[26]
	*Bacillus amylolytic*	Wheat bran	The content of NDF and ADF ↓	[27]
	*Bacillus subtilis* (KCTC 13241)	Wheat bran	The antioxidants and nutritional aspects ↑	[28]
	*Bacillus sphaericus* *Bacillus licheniformis*	Wheat bran	The content of feruloylated glycosides ↑	[29]
	*Bacillus natto*	Millet bran	The structural and functional properties of its dietary fiber ↑	[30]
	*Bacillus cereus*	Wheat bran	The content of non-starch polysaccharide ↓	[31]
	*Bifidobacterium pseudocatenulatum* (ATCC 27919)	Rye Wheat	The anti-nutritional factors content ↓	[32]
	*Bifidobacterium animalis*	Oat	The amounts of free amino nitrogen ↑	[33]
	*Enterococcus faecalis* (M2)	Wheat bran	The antioxidant capacity and free radical scavenging rate ↑	[34]
	*Pediococcus acidilactici* (M16)	Rice bran	The content of ferulic acid ↑	[20]
	*Streptococcus thermophiles*	Wheat bran	The anti-nutritional factors content ↓	[35]
Yeast				
	*Saccharomyces cerevisiae*	Rice bran	The protein content and bioactivity ↑	[36]
	*Lipomyces kononenkoae*	Sorghum	The protein content and digestibility ↑	[26]
	*Candida humilis* (E-96250)	Wheat bran	The content of total phenols ↑	[37]
	*Candida santamariae* (Y11)	Rice	The hardness, chewiness and mouthfeel of noodles ↑	[38]
Fungi				
	*Aspergillus niger*	Wheat bran	The antioxidant and anti-inflammatory capacity ↑	[39]
	*Aspergillus oryzae*	Wheat	The phenolics and free radicals scavenging activity ↑	[40]
	*Aspergillus awamorinakazawa*	Wheat	The antioxidant properties ↑	[41]
	*Rhizopus oligosporus*	Rice bran	The phenolic acid content and antioxidant activity ↑	[42]
	*Rhizopus oryzae*	Rice bran	The content of total phenolic ↑	[43]
	*Rhizopus azygoporus* (MTCC 10195)	Millet	The content of total phenolic ↑	[44]
	*Monascus Purpureus*	Rice bran	The phenolic acid content and antioxidant activity ↑	[42]
	*Monascus pilosus* (KCCM60084)	Rice bran	The content of total flavonoid ↑	[45]
	*Eurotium cristatum*	wheat bran	The soluble dietary fiber content ↑	[46]
	*Trichoderma pseudokoningii*	Wheat bran	The sugar content ↓	[47]
	*Thamnidium elegans* (CCF 1456)	Maize	The content of total phenolic ↑ The radical scavenging capacity ↑	[48]

↑: Improved or increased; ↓: Decreased.

**Table 2:** Cereal fermentation studies and the main outcomes obtained.

Target Object	Microorganism Involved in Fermentation	Fermentation Type	Fermentation Conditions	Modification(s) in Nutritional Constituents	References
Wheat					
	Spontaneous fermentation	Liquid	96 h at 30 °C	Starch granule surface was eroded; Pasting properties of wheat starch were changed.	[57]
	*Lactobacillus plantarum* *Saccharomyces cerevisiae*	Solid	2 h at 20 °C	The continuity of the gluten network was enhanced.	[58]
	*Bacillus subtilis* (KCTC 13241)	Liquid	72 h at 25 °C	The content of free phenolic compounds increased; The free radical scavenging improved.	[28]
	Spontaneous fermentation	Liquid	72 h at 20 °C	The content of tannin decreased from 0.58 mg/100 g to 0.47 mg/100 g, and the phytic acid content decreased from 464.1 mg/100 g to 371.28 mg/100 g.	[59]
Wheat bran					
	*Lactobacillus brevis* (E-95612) *Candida humilis* (E-96250)	Liquid	24 h at 30 °C	The content of free amino acids increased.	[37]
	*Lactobacillus bulgaricus Streptococcus thermophiles* Commercial baker’s yeast	Solid	24 h at 37 °C	The content of phytic acid levels reduced by 27.34%.	[35]
Rice					
	*Lactobacillus fermentum* (M9) *Candida santamariae* (Y11)	Liquid	32 h at 30 °C	The ordered structures of rice and starch granules were disrupted.	[38]
	Yeast *Lactobacillus*	Liquid	3 h at 37 °C	The contents of total starch and amylose increased.	[60]
	Commercial baker’s yeast	Liquid	6 h at 32 °C	The extractable total phenols content increased by 13%; The content of phytic acid decreased by nearly 41%.	[61]
Rice bran					
	*Rhizopus oryzae*	Solid	120 h at 30 °C	The content of ash, dietary fiber, protein, and amino acid increased. The content of water content, lipid, and phytic acid decreased.	[62]
	*Rhizopus oryzae*	Solid	72 h at 30 °C	Trypsin inhibitor activity decreased by 24.8% and phytic acid activity decreased by 3.12%.	[63]
Maize					
	*Lactobacillus casei* *Lactobacillus fermentum* *Lactobacillus plantarum*	Solid	120 h at 37 °C	Fermentation hydrolyzed part of the corn and flour had smaller, irregularly shaped particles.	[64]
	*Thamnidium elegans* (CCF 1456)	Solid	144 h at 25 °C	The content of total phenolic increased; The activity of β-glucosidase was enhanced; The radical scavenging capacity improved.	[48]
	Lactic Acid Bacteria	Solid	120 h at 25 °C	The majority of starchy compounds decreased; A slight increase of the crude proteins content; A significant increase in minerals; The tannin content reduced.	[65]
Barley					
	Spontaneous fermentation	Liquid	72 h at 35 °C	Grains with more pores, broken and cracked.	[66]
	*Lactobacillus plantarum* (dy-1)	Liquid	24 h at 31 °C	The proportion of essential amino acids increased.	[67]
	*Lactobacillus plantarum* (dy-1)	Liquid	24 h at 31 °C	The structure of β-glucan could be changed; The molecular weight of β-glucan was reduced.	[68]
	*Lactobacillus plantarum* (P-S1016)	Liquid	36 h at 37 °C	The free phenolic content + increased from 4.87 mg GAE/g to 5.61 mg GAE/g.	[23]
	*Lactobacillus johnsonii* (LA1), *Lactobacillus reuteri* (SD2112), *Lactobacillus acidophilus* (LA-5)	Liquid	18 h at 37 °C	The content of free phenolic acids increased from 2.55 to 69.91 μg/g DM.	[25]
Sorghum					
	Spontaneous fermentation	Liquid	192 h at 30 °C	The content of sorghum amylose was increased; The gelatinization temperature of sorghum starch decreased.	[69]
	Lactic Acid Bacteria	Liquid	8 h at 37 °C	Both the essential and non-essential amino acids increased; The antinutritional factors decreased	[70]
	*Saccharomyces cerevisiae* *Lactobacillus amylovorus* (NRRL B-4540) *Amylolytic* yeasts	Liquid	24 h at 35 °C	Both the phytic acid content and the ratio of phytic acid to protein decreased; The digestibility of sorghum protein was improved.	[26]
Millet					
	*Lactobacillus plantarum* (LP60171)	Liquid	96 h at 37 °C	The distribution of amylopectin chain length tended to be short chain; Fermentation improved the properties of starch.	[71]
	*Rhizopus azygoporus* (MTCC 10195)	Solid	24 h at 25 °C	The total phenolic content was enhanced from 6.6 to 21.8 mg.	[44]
	Spontaneous fermentation	Liquid	72 h at 28 °C	The content of essential and non-essential amino acids increased; The fermented biscuits had higher moisture levels, crude protein, crude fiber and energy value with a lower fat and ash content.	[72]
Oat					
	*Lactobacillus acidophilus* (LA-5), *Lactobacillus johnsonii* (LA1), *Lactobacillus reuteri* (SD2112)	Liquid	18 h at 37 °C	The content of free phenolic acids increased from 4.13 to 109.42 μg/g DM.	[25]
	*Lactobacillus plantarum* (B1-6), *Rhizopus oryzae*	Solid	72 h at 30 °C	The soluble protein contents changed from 7.05 mg/g to 14.43 mg/g for the co-inoculated fermented oats.	[73]

## Data Availability

The data presented in this study are included in the article.
